# Emergency decompression for patients with ureteral stones and SIRS: a prospective randomized clinical study

**DOI:** 10.1080/07853890.2023.2169343

**Published:** 2023-03-07

**Authors:** Xiaofei Lu, Benzheng Zhou, Dechao Hu, Yanting Ding

**Affiliations:** a Department of Urology, Xiang Yang No. 1 Peoples Hospital Affiliated Hospital of Hubei University of Medicine, Xiangyang, China; b Department of Gynaecology and Obstetrics, Xiang Yang Central Hospital, Affiliated Hospital of Hubei University of Arts and Science, Xiangyang, China

**Keywords:** Percutaneous nephrostomy, retrograde ureteral stent insertion, emergency decompression, ureteral stone, urosepsis

## Abstract

**Objective:**

Patients with ureteral calculi and systemic inflammatory response syndrome (SIRS) often require emergency drainage, and percutaneous nephrostomy (PCN) and retrograde ureteral stent insertion (RUSI) are the most commonly used methods. Our study aimed to identify the best choice (PCN or RUSI) for these patients and to examine the risk factors for progression to urosepsis after decompression.

**Methods:**

A prospective, randomized clinical study was performed at our hospital from March 2017 to March 2022. Patients with ureteral stones and SIRS were enrolled and randomized to the PCN or RUSI group. Demographic information, clinical features and examination results were collected.

**Results:**

Patients (*n* = 150) with ureteral stones and SIRS were enrolled into our study, with 78 (52%) patients in the PCN group and 72 (48%) patients in the RUSI group. Demographic information was not significantly different between the groups. The final treatment of calculi was significantly different between the two groups (*p* < .001). After emergency decompression, urosepsis developed in 28 patients. Patients with urosepsis had a higher procalcitonin (*p* = .012) and blood culture positivity rate (*p* < .001) and more pyogenic fluids during primary drainage (*p* < .001) than patients without urosepsis.

**Conclusion:**

PCN and RUSI were effective methods of emergency decompression in patients with ureteral stone and SIRS. Patients with pyonephrosis and a higher PCT should be carefully treated to prevent the progression to urosepsis after decompression.

**Key message**
- In this study, we evaluate the best choice (PCN or RUSI) for patients who have ureteral stones and SIRS and to examine the risk factors for progression to urosepsis after decompression. This study found that PCN and RUSI were effective methods of emergency decompression. Pyonephrosis and higher PCT were risk factors for patients to develop to urosepsis after decompression.

## Introduction

Systemic inflammatory response syndrome (SIRS) is a severe complication of acute obstructive pyelonephritis (AOP) that is caused by ureteral stones before urosepsis develops. Ureteral stone-related AOP is a urological emergency because it can cause a rapid loss of renal function and quickly develop to urosepsis or even septic shock within hours [1]. Once urosepsis or septic shock develops, the mortality rate is high, and 2%–9% patients will die [1–3]. Thus, earlier emergency decompression is needed for patients who have ureteral stones and SIRS to reduce the absorption of toxic substances or bacteria and prevent the progression to urosepsis.

Percutaneous nephrostomy (PCN) and retrograde ureteral stent insertion (RUSI) are the two most commonly used emergency drainage methods, and the more effective method remains controversial. Tambo et al. [3] conducted a retrospective study of 69 patients with acute obstructive pyelonephritis, and they found that PCN and RUSI are both effective surgical decompression methods, and Anıl et al. [4] came to the same conclusion. However, Mokhmalji et al. [5] and Xu et al. [6] found that PCN is superior to RUSI for patients with urosepsis caused by acute upper urinary tract obstruction. Previous studies all had a limited number of patients, and the patients had progressed to sepsis before emergency drainage was performed. There are no reports on how to manage calculi after decompression. Here, we performed a prospective, randomized, larger-scale case study to evaluate the best choice (PCN or RUSI) for patients who have ureteral stones and SIRS and to examine the risk factors for progression to urosepsis after decompression.

## Materials and methods

### Patient enrolment

**Figure 1.**
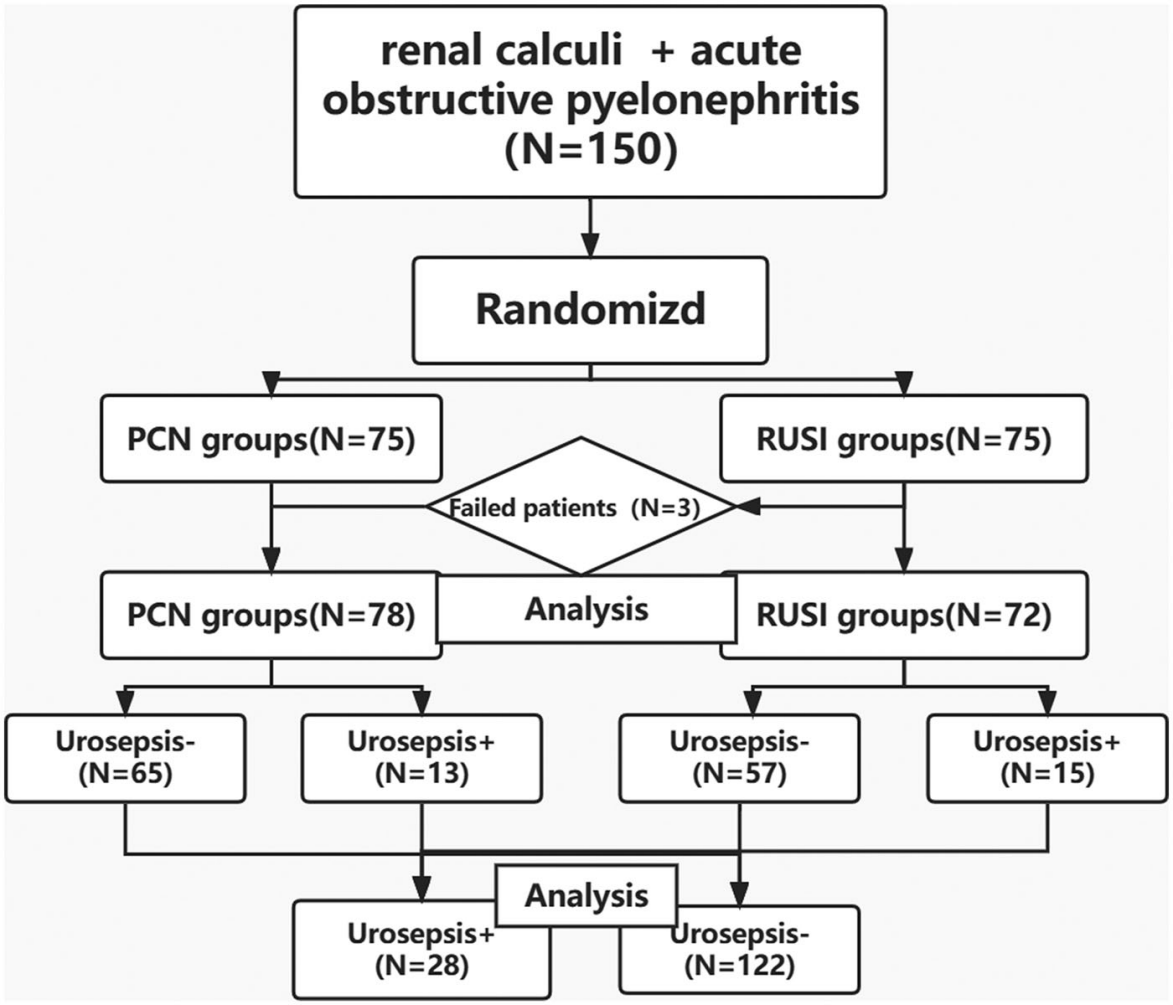
Flow chart of the clinical study.

From March 2017 to March 2022, patients with ureteral stones and SIRS were randomized into the PCN group (*n* = 75) or the RUSI group (*n* = 75) (Figure 1). In the RUSI group, three patients were moved into the PCN group after retrograde ureteral stent failure. Finally, there were 78 patients in the PCL group and 72 patients in the RUSI group.

### Inclusion and exclusion criteria

The inclusion criteria were patients diagnosed with ureteral stones and SIRS at admission and patients with complete clinical information. The patients were diagnosed with ureteral calculi based on imaging examinations, such as ultrasonography or computed tomography (CT). SIRS was diagnosed based on the presence of two of the following symptoms: hyperpyrexia (body temperature >38.0 °C) or hypothermia (body temperature <36.0 °C); tachycardia (heart rate, >90/min); tachypnoea (>20 breaths/min or PaCO_2_ <32 mmHg); and white blood cell (WBC) count >12,000/mm^3^ or <4000/mm^3^ or immature cells >10%. Urosepsis was defined as a Quick Sepsis-related Organ Failure Assessment (qSOFA) score of 2 or higher [7].

The exclusion criteria were as follows: patients with infectious diseases in other parts of the body or who received long-term immunosuppressive therapy; patients who had bilateral ureteral stones or coagulation disorders or patients with abnormal urinary tract anatomy.

### Therapeutic process

A mid-stream urine culture (MUC) and a blood culture were performed after hospitalization, and empiric antibiotic therapy (cephalosporin) was administered because *Escherichia coli* is the most common bacteria that causes urinary tract infections. According to the results of bacterial culture and drug sensitivity test, the patients were switched to treatment with sensitive antibiotics. Emergency compression was conducted using PCN or RUSI, and the emergency compression drainage fluid was also cultured. The patient’s temperature was monitored continuously until it returned to normal (<37.3 °C).

In the PCN group, patients were placed into the prone position, and the renal puncture point was located using ultrasound. Under local anaesthesia, the nephrostomy catheters (8 Fr) was inserted under ultrasound guidance and drainage fluid was obtained for bacterial culture. Emergency ultrasound or CT was performed to check whether drainage was restored. If failed, the patients were switched to the RUS group in time.

In the RUSI group, patients were placed into the lithotomy position. Urethral surface anaesthesia was performed, and a zebra guidewire was inserted into the ureter on the affected side using a cystoscope. A F5 DJ tube was inserted and guided by a guidewire. Then, we conducted catheterization for all the DJ patients and a urine sample was taken from the catheterization during DJ stent insertion. An emergency ultrasound or CT was performed to check whether drainage was restored. If failed, the patients were switched to the PCN group in time.

### Clinical data collection and statistical analysis

We estimated that a total of 150 patients would be needed to compare a difference between groups, with a two-tailed α of 0.05 and a (1 − β) of 0.90. Our initial estimate of same size included an assumption non-compliance of 20%.

Patients’ clinical data were collected including WBCs, neutrophils, serum C-reactive protein (CRP), PCT, urine leukocytes, urine culture, serum creatinine, stone size, stone density, hydronephrosis grade, symptom duration, nature of drainage fluid, length of hospital stay (days) and time for body temperature to return to normal (days). A Student’s *t*-test or Mann–Whitney U test was performed for continuous data, and a Chi-square test or Fisher exact test was performed for categorical data. Statistical analysis was performed using SPSS version 26 (IBM Corp., Armonk, NY, USA), and *p* < .05 was considered to represent a significant difference.

## Results

One hundred fifty patients with ureteral stones and SIRS were enrolled into our study from March 2017 to March 2022. Patients were randomized into the PCN group or the URI group, and among patients in the URI group, three patients were switched to the PCN group after retrograde ureteral stent failure. Finally, 78 (52%) patients in the PCN group and 72 (48%) patients in the RUSI group were analysed. The success rate of drainage in the PCN group was 100% and that in the URI group was 96%.

Patients data before decompression were analysed in both groups, and there were no significant differences between the two groups (*p* > .05; Table 1). After decompression, there was a marked decrease in WBC, neutrophils, serum creatinine, serum CRP and PCT levels in all patients, but there were no statistically differences between the two groups (*p* > .05; Table 2). Surgical duration in the PCN and URSI groups was similar (23.55 ± 5.02 min vs. 24.27 ± 4.99 min). The time for temperature to return to normal appeared to be shorter in the PCN group (2.35 ± 0.58 day vs. 2.51 ± 0.62 day), but the difference was not statistically significant (*p* = .072). There was also no significant difference between the two groups in the visual analogue scale score and length of hospital stay. However, the final calculi treatment was significantly different between the two groups (*p* < .001). Additionally, more patients in the URSI group compared with the PCN group were treated with ESWL (21 vs. 5) after decompression.

**Table 1.** Basic data of patients.

	PCN	RUSI	*p* Value
Patients (%)	78 (52)	72 (48)	
Age (years)^a^	53.76 ± 11.24	56.40 ± 10.56	.994
Sex (male/female)^b^	52/26	47/25	.599
Body mass index(BMI)^c^	22.45 ± 2.31	22.52 ± 1.95	.146
Associated co-morbid condition(n/N)			
Diabetes mellitus^b^	20/78	14/72	.365
Hypertension^b^	38/78	34/72	.855
Duration of symptom (day)^a^	3.28 ± 2.19	2.75 ± 2.14	.763
Stone size (mm)^c^	10.15 ± 4.26	9.88 ± 4.77	.869
Stone density (HU)^c^	872.26 ± 406.4	813.00 ± 354.42	.367
Grade of hydronephrosis (n/N)^b^			.560
Grade 1/2	47	40	
Grade 3/4	31	32	
WBC (*10^9/L)^a^	13.61 ± 5.69	15.10 ± 6.07	.663
Neutrophils (*10^9/L)^a^	10.50 ± 5.12	11.95 ± 6.32	.073
Serum CRP (mg/L)^a^	96.78 ± 74.53	108.82 ± 76.32	.887
Serum albumin^c^	35.22 ± 6.68	36.95 ± 5.41	.179
PCT^a^	8.79 ± 11.61	8.19 ± 11.07	.593
Serum creatinine^a^	118.64 ± 86.95	151.25 ± 117.85	.271
Urine leukocyte (/ul)^a^	124.02 ± 333.77	158.46 ± 404.81	.241
Urine culture^b^			.880
Positive	20	16	
Negative	58	56	
Blood culture^b^			.817
Positive	23	28	
Negative	55	44	

*Note:* PCN: percutaneous nephrostomy; RUI: retrograde ureteral intubation; WBC: white blood cells; CRP: C-reactive protein; PCT: procalcitonin.

^a^Mann–Whitney U test.

^b^Chi square test.

^c^Student’s t-test.

**Table 2.** Clinical data after emergency decompression.

	PCN	RUSI	*p* Value
Patients (%)	78 (52)	72 (48)	
Post-WBC (*10^9/L)^a^	6.95 ± 2.73	7.55 ± 3.11	.188
Post-Neutrophils (*10^9/L)^a^	5.64 ± 3.63	6.62 ± 3.37	.236
Post-Serum CRP (mg/L)^a^	59.89 ± 52.91	71.2 ± 52.73	.824
Post-PCT^a^	71.2 ± 52.73	72.14 ± 2.42	.676
Post-Serum creatinine^a^	85.42 ± 46.89	95.61 ± 69.46	.099
Temperature return to normal(day)^c^	2.35 ± 0.58	2.51 ± 0.62	.072
Length of stay (day)^c^	7.92 ± 2.49	8.37 ± 3.07	.215
Operation time (min)^c^	23.55 ± 5.02	24.27 ± 4.99	.834
Definitive treatment (PCNL or URL/ESWL)^b^	73/5	51/21	<.001*
VAS scores^c^	4.62 ± 1.74	4.52 ± 1.54	.277

*Note:* *p < 0.05. ESWL: extracorporeal shock wave lithotripsy; VAS: visual analogue scale.

^a^Mann–Whitney U test.

^b^Chi square test.

^c^Student’s t-test.

The risk factors for urosepsis after emergency decompression were analysed (Table 3). There was no significant difference between the two groups in primary drainage after PCN or RUSI and basic characteristics. There was a statistically significant difference in PCT (*p* = .012), blood culture (*p* < .001), nature of drainage fluid (*p* < .001) between the two groups. Patients with urosepsis had a higher PCT, a higher positive blood culture rate and more pyogenic fluid during primary drainage than patients without urosepsis (28.09 ± 12.12 vs. 7.15 ± 3.86; 67.9% vs. 16.4%; 71.4% vs. 28.6%, respectively).

**Table 3.** Factors of urosepsis after emergency decompression.

	Urosepsis(−)	Urosepsis (+)	*p* Value
Patients (%)	122 (81.3)	28 (18.7)	
Age (years)^a^	54.88 ± 11.04	57.00 ± 9.87	.594
Sex (male/female)^b^	80/42	19/9	.818
Body mass index^c^	22.64 ± 2.41	22.33 ± 1.85	.223
Associated co-morbid condition (n/N)			
Diabetes mellitus^b^	24/122	10/28	.067
Hypertension^b^	58/122	14/28	.814
Duration of symptom (day)^c^	3.12 ± 2.18	2.61 ± 2.15	.625
Stone size (mm)^c^	9.18 ± 4.59	10.00 ± 2.84	.062
Stone density (HU)^a^	835.18 ± 382.48	881.46 ± 385.74	.889
Grade of hydronephrosis (n/N)^b^			.599
Grade ½	72/122	15/28	
Grade ¾	50/122	13/28	
WBC (*10^9/L)^a^	13.24 ± 5.03	21.59 ± 5.03	.255
Neutrophils (*10^9/L)^a^	9.73 ± 4.83	17.90 ± 5.01	.252
Serum CRP (mg/L)^a^	95.93 ± 77.33	95.93 ± 77.33	.131
Serum albumin^c^	34.60 ± 6.44	34.45 ± 4.41	.107
Serum creatinine^a^	134.26 ± 104.67	154.61 ± 98.39	.485
PCT^a^	7.15 ± 3.86	28.09 ± 12.12	.012*
Urine leukocyte (/ul)^a^	130.72 ± 362.12	180.53 ± 547.35	.171
Urine culture^b^			.731
Positive	28 (22.9%)	8 (28.6%)	
Negative	94 (81.1%)	20 (71.4%)	
Blood culture (%)^b^			<.001*
Positive	32 (26.2%)	19 (67.9%)	
Negative	90 (73.8%)	9 (32.1%)	
Nature of drainage fluid(%)^b^			<.001*
Pyogenic fluids	32 (26.2%)	20 (71.4%)	
Nonpyogenic fluids	90 (73.8%)	8 (28.6%)	
PCN/RUSI^b^	66/56	12/16	.283

*Note:* *p < 0.05. PCN: percutaneous nephrostomy; RUSI: retrograde ureteral stent insertion; WBC: white blood cells; CRP: C-reactive protein; PCT: procalcitonin.

^a^Mann–Whitney U test.

^b^Chi square test.

^c^Student’s t-test.

Fifty-one (34%) patients had a positive blood culture result, and the most common causes of the urinary tract infection were *E. coli* (66.7%), *Klebsiella pneumoniae* (15.7%) and *Enterococcus faecium* (9.8%). There were no significant differences in the pathogenic bacteria between the two groups (Table 4).

**Table 4.** Pathogenic bacteria of patients.

Variables	Total	Urosepsis (−)	Urosepsis (+)	*p* Value
Positive blood culture^a^	51 (34%)	32 (26.2%)	19 (67.8%)	<.001*
*Escherichia coli* ^b^	34 (66.7%)	20 (39.2%)	13 (25.5%)	.669
*Klebsiella pneumoniae* ^b^	8 (15.7%)	5 (9.8%)	4 (7.8%)	.685
*Enterococcus faecium* ^b^	5 (9.8%)	4 (7.8%)	1 (1.9%)	.401
Candida^b^	3 (5.9%)	2 (3.9%)	1 (1.9%)	.885
*Proteus* spp.	1 (1.9%)	1 (1.9%)	0	

^a^Chi-square test.

^b^Fisher exact test.

*p < 0.05.

## Discussion

The main finding of this study is that PCN and RUSI are effective and safe methods of emergency decompression for patients with ureteral stones and SIRS. There were no significant differences between the two groups in basic data and length of hospital stay of patients, and the time for temperature to return to normal appeared to be shorter in the PCN group, but the difference was not statistically significant. Additionally, we found patients with pyonephrosis and a higher PCT are more likely to progressed to urosepsis after early emergency drainage.

Before urosepsis develops, the timing of when emergency drainage is performed is crucial; most experts agree that emergency drainage should be performed within two days of admission [2,4,8–10]. Blackwell et al. found that, compared with early decompression (within 48 h after admission), delayed decompression significantly increases the mortality rate of patients with acute obstructive pyelonephritis caused by ureteral stones [9]. Kozyrakis also found that one-third of patients developed urosepsis after decompression that was performed beyond 48 h after admission. They suggested that emergency drainage within 48 h after admission can significantly reduce the patients’ mortality rate [2]. Similarly, Nishiguchi et al. compared the length of hospital stay in the early stenting group to that of the delayed stenting group, and they found that early stenting within 48 h can significantly reduce the length of hospital stay in these patients [10]. SIRS is considered to be a warning sign for urosepsis [1]. In our study, over 80% of patients developed SIRS within two days after admission, and emergency drainage was performed in most patients within 48 h of admission. Twenty-eight (18.7%) patients developed urosepsis after decompression, and none of these patients died. Thus, we suggest that when SIRS occurs, emergency drainage should be performed as soon as possible.

PCN and RUSI are the most commonly used methods for emergency drainage, and identifying the more effective of these two methods has been controversial. A prospective, randomized study was recently performed to compare the best treatment for patients with urosepsis caused by ureteral stones. These researchers found that PCN was superior to RUSI for emergency drainage [6]. Mokhmalji et al. also found that the PCN success rate was obviously higher than that of RUSI (100% vs. 80%) [11]. Other studies suggested that retrograde double J tube placement can cause severe urethral pain during an endoscopic procedure in men [10]. With the development of ureteroscopy, the RUSI success rate was up to 98%, and urinary tract pain was less severe [12]. Tambo et al. found that both PCN and RUSI are effective surgical decompression methods, and they suggested that RUSI was less invasive and had a lower risk of haemorrhage than PCN [3].

In our study, the PCN success rate was similar to RUSI (100% vs. 96%), and PCN and RUSI are effective methods of emergency decompression for patients with ureteral stones and SIRS. Inflammatory indicators after drainage and the length of hospital stay were also compared between the two groups, and there were no statistically significant differences. The time for the temperature to return to normal seemed to be shorter in the PCN group compared to the RUSI group (2.35 ± 0.58 vs. 2.51 ± 0.62 days), but this difference was not statistically significant. For the definitive treatment, more patients in the URI group than in the PCN group chose ESWL (21 vs. 5) for their definitive treatment, and the chance of having a second surgery was significantly reduced the URI group, To the best of our knowledge, this was the first article to introduce further treatment of patients with ureteral stones and SIRS after emergency drainage. Therefore, we believe that RUSI is superior to PCN in reducing the probability of secondary surgery for patients with a lower stone load.

There are many risk factors for urosepsis or septic shock. Previous studies showed that female sex, older age, diabetes mellitus, extended-spectrum ß-lactamase-producing (ESBL)-positive *E. coli*, severe hydronephrosis, thrombocytopenia, hypoproteinaemia and immunosuppression are independent risk factors for urosepsis [1–3,13–17]. Patients with risk factors often require emergency drainage to prevent urosepsis progression [2,3,6]. However, some patients developed urosepsis and required intensive care even with timely drainage. In this study, 28 (18.7%) patients developed urosepsis after decompression, but none of them died because the early emergency drainage and intensive management in the intensive care unit at our hospital were successful. We found that the serum procalcitonin (PCT) in the urosepsis group was higher than in the no urosepsis group (28.09 ± 12.12 vs. 7.15 ± 3.86; *p* = .012). PCT is a precursor protein with 116 amino acids that is synthesized and secreted by thyroid C cells [18]. When infection or trauma occurs, PCT levels *in vivo* increased significantly within 12 to 48 h and remained stable. The PCT level can reflect disease severity [19]. A previous study showed that PCT is a tool for early diagnosis and monitoring of urosepsis after percutaneous nephrolithotomy [19]. Similarly, Cui et al. found that the PCT level can accurately predict urosepsis development [20]. Our data also suggest that PCT is an risk factor for patients with urosepsis after decompression.

The nature of drainage fluid is another risk factor for patients with urosepsis. Pyonephrosis was found in 20 of 28 patients with urosepsis during primary drainage. The evolution of renal pyonephrosis has not been investigated. Obstruction caused by ureteral stones and a bacterial infection are two types of primary pathogenesis [21].

Boeri et al. also showed that pyonephrosis can cause a rapid loss of renal function and quickly develop into urosepsis or even septic shock [22]. Thus, emergency decompression is required to protect renal function and prevent urosepsis development in patients with pyonephrosis. In this study, we found that pyonephrosis is a risk factor development of urosepsis after decompression (*p* < .001).

Infection is another requirement. *E. coli* is the most common infectious organism in urinary tract infections [23,24]. Similar results were also found in this study, where 34 (66.7%) patients were infected with *E. coli* among all patients with positive cultures. In a previous study, Huang et al. suggested that ESBL *E. coli* was an independent risk factor for urosepsis [23]. However, ESBL was not observed in our study, and thus, we did not find that *E. coli* is a risk factor for patients developing urosepsis after decompression (*p* = .669).

MUC and blood culture are the most commonly used methods to identify pathogens. However, some experts believe the MUC cannot be a good predictor of urosepsis because of its low positive rate [24,25]. In a previous study, the positive MUC rate in patients with pyonephrosis was below 50% [24]. Liu et al. also found that the infection may persist in the upper system when the MUC is negative due to the obstruction caused by the ureteral stone [26]. In this study, the positive MUC rate is 24% in patients with ureteral stones and SIRS, and we found that MUC is not a risk factor for patients developing urosepsis after decompression (*p* = .731). However, blood culture results seem to be a good predictor of urosepsis. In the literature, a positive blood culture result is the gold standard for confirming urosepsis [27]. In this study, 32 patients had a positive blood culture result and did not develop urosepsis, but there was a significant difference between patients with or without urosepsis (*p* < .001). Our data suggest that a positive blood culture result is a risk factor for the development of urosepsis after decompression.

### Limitations of study

There were some limitations in our study. First, only the qSOFA score was used in this paper, and the SOFA criteria were not used for further diagnosis. Second, emergency drainage was performed in some patients within 24 h after admission. Early drainage may not be necessary in some patients and may be over-treatment for patients with a partial obstruction or whose obstruction can be controlled using empiric antibiotic therapy. Finally, this was a single-centre study, and the sample size was still relatively small. A large-scale multi-centre analysis with a larger sample size is required to confirm our conclusions.

## Conclusion

Our data suggest that PCN and RUS are effective methods of emergency decompression for patients with ureteral stones and SIRS. However, RUSI was superior to PCN in reducing the probability of secondary surgery for patients with a lower stone load. Thus, we suggest that RUSI should be used for emergency drainage in patients with small ureteral calculi. Patients with pyonephrosis and a higher PCT should be carefully treated to prevent progression to urosepsis after decompress.

## Data Availability

All data generated or analysed during this study are included in this article. Further enquiries can be directed to the corresponding author.
